# Prioritization of interventions in pursuit of maternal health policy objectives to mitigate stillbirth risks. An exploratory qualitative study at subnational level in Uganda

**DOI:** 10.1186/s12913-020-06046-z

**Published:** 2021-01-11

**Authors:** Eric Ssegujja, Isaac Ddumba, Michelle Andipartin

**Affiliations:** 1 Makerere University School of Public Health, Kampala, Uganda; 2 University of the Western Cape, School of Public Health, Cape Town, South Africa; 3 Mukono District Local Government, Mukono Town, Uganda; 4 Department of Psychology, University of the Western Cape, Cape Town, South Africa

**Keywords:** Stillbirth, Policy translation, Subnational level, Service delivery, Health workers

## Abstract

**Background:**

Global calls for renewed efforts to address stillbirth burden highlighted areas for policy and implementation resulting in national level translations. Information regarding adapted strategies to effect policy objectives into service delivery by frontline health workers remains scanty especially at subnational level. The study explored strategies prioritized to mitigate stillbirth risk in the context of operationalizing recommendations from the global campaigns at a subnational level in Uganda.

**Methods:**

A cross-sectional qualitative exploratory study was conducted among a purposively selected sample of sixteen key informants involved in delivery of maternal and child health services in Mukono district. Analysis followed thematic content analysis deductively focusing on those policy priorities highlighted in the global stillbirth campaigns and reflected at the national level in the different guidelines. *Results.*

Interventions to address stillbirth followed prioritization of service delivery aspects to respond to identified gaps. Efforts to increase uptake of family planning services for example included offering it at all entry points into care with counseling forming part of the package following stillbirth. Referrals were streamlined by focusing on addressing delays from the referring entity while antenatal care attendance was boosted through provision of incentives to encourage mothers to comply. Other prioritized aspects included perinatal death audits and improvements in data systems while differentiated care focused on aligning resources to support high risk mothers. This was in part influenced by the limited resources and skills which made health workers to adapt routine to fit implementation context.

**Conclusions:**

The resource availability determined aspects of policy to prioritize while responding to stillbirth risk at subnational level by frontline health workers. Their understanding of risk, feasibility of implementation and the desire for optimal health systems performance worked to define the nature of services delivered calling for purposeful consideration of resource availability and implementation context while prioritizing stillbirth reduction at subnational level.

**Supplementary Information:**

The online version contains supplementary material available at 10.1186/s12913-020-06046-z.

## Background

Close to 2.6 million stillbirths occur every year. Most of the cases happen in Low and Middle-Income Countries (LMICs) [1, 2] with rural areas disproportionately affected. Addressing the burden attracted global campaigns with country targets of 14/1000 live births by 2030 [3]. Policy options included adoption of best buys at global level followed by national level translation [4] for sustainability [5]. The 2011 Lancet Stillbirth series “call to action” urged countries to ensure that stillbirths are reflected in maternal and newborn health plans [6] by linking stillbirth reduction to maternal and newborn care [7] with specific time-bound goals. Proposed actions included collection and reporting of accurate stillbirth rates, causes of death, auditing of stillbirths, actions to reduce the stigma among others [8].

National level strategies around prenatal, antenatal and postnatal care [4] alongside health systems strengthening follow an integrated approach with experiences from elsewhere suggesting that implementation is affected by contextual complexities hence affecting results [9]. Uganda embraced themes from the global stillbirth campaigns with some integrated into the National Reproductive Maternal, Newborn and Child Health policies [10]. However, information regarding extent of implementation at subnational level remains scanty. We sought to understand the extent to which the frontline health workers influenced the implementation of interventions adopted from the global stillbirth campaigns into national priorities.

The nonlinear nature of policy implementation processes [11] means that frontline health workers are critical in determining implementation success [12]. In resource limited settings where almost 98% of the global stillbirth burden occurs [9], maternal and child health services are mainly delivered by nurses/midwives who form the bulk of the health workforce. Inability to achieve policy objectives becomes chronic. With inadequate support supervision from the centre to ensure compliance, interpretation of policy intentions becomes subject to individuals’ understanding. Harmonization with available resources determines what finally gets delivered. Frontline health workers hence become key to the success of policy translation influenced by the discretionary powers they wield on a case by case basis [13]. Such a role casts them to determine implementation dose depending on the resources available.

## Methods

### Design

This study adopted a cross-sectional qualitative design to address sub-national health systems perspectives related to frontline health worker’s translation of interventions to respond to stillbirth burden at that level.

### Setting

The study was conducted in Mukono district following criteria informed by subnational level performance derived from the Annual Health Sector Performance Report (AHSPR) 2015/16 where district comparisons in terms of stillbirth burden are computed among other health indicators. At the time of conceptualizing this study, it was ranked in the second tier of districts with high stillbirth rates ranging between 9 and 14.9/1000 live births with malaria as the leading cause of morbidity and mortality. Located in the central region of the country, the population was estimated at 599,817 of which 307,927 were female and ranked the 7th among the most populated district in Uganda. Of the 14,240 deliveries conducted at the health facilities, 240 were stillborn of which 112 were fresh stillbirths (District Health report 2016/17) and yet less than half (49.4%) of deliveries were conducted by public sector facilities where data is routinely collected. A number of the known stillbirth risk factors were common in the study area, for example, malaria rates during pregnancy at 42.4%, early initiation of childbearing and early marriages with a high ideal number of children. The district had a fully constituted District Health Management Team (DHMT) with four health sub-districts. The characteristics of a pluralistic system are evident with a relatively fair distribution of public, private not for profit health facilities and a heavy presence of the private-for-profit health facilities. The community component is served with a Community Health Worker structure known as the Village Health Team (VHTs) and Civil Society Organizations (CSO) implementing health-related interventions. The district is categorized as hard to reach by Ministry of Health (MoH) because of several islands stretching into Lake Victoria. It was one of the districts where the national level rollout of Helping Babies Breath (HBB) and HBB plus (an evidence-based capacity building program to teach neonatal resuscitation techniques in resource limited settings) were piloted. It, therefore, presented a unique and interesting case to understand how frontline health workers influenced the translation of global campaign strategies to address stillbirth through the implementation of policy guidelines at the subnational level.

### Participants characteristics

Six health facilities were selected for an in-depth case study in addition to the District Health Management Team (DHMT) purposively selected based on the level of service provision and ownership. Face to face interviews with managers and frontline health workers directly involved in clinical and managerial decision making were conducted. A list of all health facilities was obtained from the annual district health report from where respondents were purposively selected on top of district health managers and sub-district health managers. A total of sixteen respondents were purposively selected where at the DHMT level, the respondents doubled in more than one role., For example, the head of the health sub-district is by mandate a members of the DHMT and may be the head of one of the health facilities. The same applied to some of the midwives interviewed where they doubled as maternity unit in-charges. The inclusion criteria were specific to respondents whom through their clinical practice or managerial functions influenced decisions related to addressing stillbirth burden. Details of respondent characteristics are in the Table 1 below.

**Table 1:** Respondent characteristics

Cadre	Level of service provision				Total
	DHMT	Hospital	HCIV	HCIII	
Medical officer	1	2	2		**5**
Nurse	2	2	1		**2**
Midwife		2	2	2	**9**
**Total**	**3**	**6**	**5**	**2**	**16**

### Description of the process

Contacts were initiated with the District Health Team (DHT) while introducing the study from where discussions about potential respondents were raised. A list of health facilities was shared and from each of the facility, a potential respondent would be identified. At the facility level, after introducing the study, further consultations regarding the participation of other staff were conducted besides the one identified from the district. Finally, potential participants were contacted and the study purpose explained and a written informed consent administered before the interview. Interviews lasted approximately one hour and were conducted at the respondents’ places of work. Overall none of the contacted potential respondents declined to participate. Broadly the interview guide was developed for this study and informed by the literature (attached here as supplementary file). It contained eight questions which explored the health worker’s experiences, the role of both facility management and individual health workers in ensuring that stillbirth risk factors were responded to appropriately. It further explored the influence of the workplace context and mothers characteristics in translating guidelines at the frontline. All interviews were audio-recorded with a digital recorder and at the end of each field day, field notes were expanded and recorded data downloaded onto the computer with a copy saved on the hard drive.

### Data analysis

Analysis followed thematic content analysis deductively focusing on those policy priorities highlighted in the global stillbirth campaigns and reflected at the national level in the different guidelines. Transcriptions were typed into Microsoft office word by the first author and a research assistant who participated in data collection and thereafter entered into Atlas.ti a data management software. Textual data relating to a particular theme corresponding to the policy priorities highlighted from the global stillbirth campaign would be highlighted and attached to a code within a codebook developed by the study team. A manual process of pile sorting similar quotes attached to particular codes was conducted which led to the identification of sub-themes under each code. Summarized mini-statements from which following a perusal process, the main themes were derived. Particular quotes from transcripts were used while presenting results to emphasize specific themes and illuminate the voices of study participants. Preliminary results were discussed with two of the participants for feedback and the revisions following the incorporation of feedback are what are reported here.

## Results

### Prioritization of health service delivery to respond to identified gaps

#### Delivery of family planning services at all entry point

Health facilities continued to pursue policy provisions from the Ministry of Health despite acknowledgment that current models of delivery contributed to loopholes. Frontline health workers altered the delivery model to prioritize all women presenting for other health services. As a result, family planning services were introduced at all entry points into care. The level of integration and compliance depended on health workers’ innovativeness with variations across facilities

*these days FP is at every entry point there are FP methods and all health workers including the clinicians and nurses are taught how to deliver the services so immediately the mother is delivered there they are counselled about the FP methods* ***PF_HCIV_004_IC_Mat***

For mothers that experienced a stillbirth, Family Planning counselling formed part of the key timely interventions where they did not stop at providing emotional support and wait for mothers to return for postnatal care to introduce family planning. Depending on the circumstances surrounding the delivery, health workers used their discretion to determine whom and what method to propose to the mother and under what circumstances;

*we counselled that mother and we even advised her to first use family planning for some time. And by the time she feels like getting pregnant again, she will be ready. We sat them down together with the husband and they agreed to come back and indeed they came back after two weeks* ***PF_HCIII_005_IC_Mat***

#### Management of infections during pregnancy

To enforce MoH policy strategies targeting HIV among pregnant women, a double-check approach to track all HIV+ mothers within antenatal care (ANC) was enforced. Facilities would review records at the end of every month to identify HIV+ mothers where efforts would be initiated by that in-charge to ensure all were initiated onto Antiretroviral Therapy (ART).

*At the end of the month we even go to the antenatal register we review and identify HIV+ mothers and check whether they were started on medication and those that were not started because they were still in denial but returned after a week.* ***PF_HCIII_006_IC_Mat***

Delivery of services to prevent malaria during pregnancy was re-purposed to have it as a Directly Observed Therapy (DOT) within the facility. This was a move from earlier arrangements of provision of or recommend for buying drugs and take from home. Safe drinking water was provided to the mothers where some facilities started mothers from 12 weeks while others at 13 weeks. This was sometimes challenged by inadequate supply leading to stock-outs. Under such circumstance’s women would be asked to buy from private pharmacies.

*MoH has a program of giving them presumptive preventive treatment where we give fancida to these women. Initially, we were giving them two dosses now they tell us we should give them monthly so long as the women are 12 weeks pregnant. Each time they come for antenatal we give them a dose … … we used to tell mothers to take fancida from home but now it is on DOT even the water is there.* ***PNFP_Hosp_001_IC_Mat***

#### Improving ANC attendance

To boost antenatal care attendance, some facilities aligned resources to introduce incentive packages targeting good adherence and completion rates. A reward system based on compliance within ANC was rolled out as exemplified in the following quotation;

*we have now designed an intervention of giving out incentives to the mothers, when they come for the first ANC visit we give them a mosquito net and when they come for the second ANC we give them a leaflet that has information about labour, outcomes of labour and about the child. And when they come for the third ANC visit we give them a baby cloth and when they come for the fourth we give them a mama kit and when they deliver from here we give them an overall and a blanket for the child*. ***PNFP_Hosp_001_MO***

The referral system was re-purposed to focus on the place of origin of referrals from one level to another in a bid to improve the turnaround time. Innovative strategies were implemented focusing on referring facilities other than waiting for cases. Frontline health workers initiated communication with referring facilities not to withhold mothers for too long, encouraged to communicate with the receiving facility before referral, close supervision and supplying referring facilities with referral forms to streamline the process;

*So what they do because they have got a file for a referral or prolonged labour, ruptured uterus, our doctor who is the medical superintendent and he heads the sub-district he goes there [referring facility] and asks them what is happening. Like previously we had one from XXX HCIII so the doctor had to go there and find out but the mother had a ruptured uterus and she lost the baby so we had to tell the doctor to talk to them.* ***PNFP_Hosp_002_IC_Mat***

#### Review of perinatal deaths

In some health facilities, health workers prioritized the review of perinatal deaths to focus on case assessment with staff that handled the mother before Maternal and perinatal Death Surveillance and Review (MPDSR) committee meeting. After identification of circumstances surrounding a particular case, the review meetings would be made aware of such information. Other would brainstorm about a particular case during departmental meetings to make recommendations and agree on possible interventions to address the identified gaps. These would inform part of the content during Continuous Medical Education (CMEs) sessions.

*in case we get one, in maternity as a department we sit with the in-charge and brainstorm on what the cause could have been and when they reach in the committee they are already aware of what could have caused that.* ***PF_HCIV_003_MW***

#### Efforts to strengthen data systems

Reporting about stillbirth at health facility level was one of the performance indicators prioritized for capture in the daily handover reports from the maternity unit. This was in addition to other existing reporting such as the surveillance, monthly and perinatal death review reports,

*So you make sure you have given them indicators that they report about like the perinatal death, stillbirth, and maternal deaths. So we have given them particular things that they report about such that we can monitor what is going on* ***PNFP_Hosp_002_MW***

In particular instances, efforts to strengthen data capture were reportedly unpopular especially among grieving parents. Some mothers were noted to be unappreciative of the exercise occasioned with circumstances of rushing for burial and hesitant to provide information.

*when one sees that hers didn’t survive and the others are having their babies, she becomes desperate and by the time you think of sitting and completing this form she will just tell you that musawo [health worker] I will come back to complete this form they want to take the dead body and will pretend that they are in a rush and want to leave … ... sometimes we fail to sit with them and complete the forms* ***PF_HCIII_006_IC_Mat***

#### Strengthening health systems

Causes attributed to gap within the health systems identified during a perinatal death reviews such as inadequate health worker skills or perceived negligence acted as the trigger for facility-wide improvements. In such instances, the review extended to ensure agreed-upon actions to address the gaps are implemented without necessarily communicating to the mother in details. In some cases, detailed investigations were conducted for fresh stillbirths than a macerated stillbirth. The same was true while responding to cases emanating from delayed referrals from known facilities compared to the ones from unknown or facilities with fewer linkages to the receiving facility such as private clinics and Traditional Birth Attendants.

*we now know that if we receive a referral-in from a government health facility or private clinics. It is very important that we act very fast because we are quite sure that these mothers could have had delays from very many points.* ***PNFP_Hosp_002_MO***

### Differentiated care to match the perceived risk

Depending on resource availability, frontline health workers offered differentiated care while delivering interventions to respond to stillbirth risks depending on the magnitude of the risk. In four of the six health facilities, all presumed high-risk mothers were referred to Doctors for management at tertiary facilities while the ones perceived at low risk continued receiving services from midwives.

*if a mother comes with hypertension, previous fresh scar diabetes, they are always referred to the doctor and they are always seen after 2 months depending on the treatment and the degree of the case.* ***PNFP_Hosp_002_MW***

#### Individualized ANC and delivery package for pregnant women

During antenatal care, health workers based on the assessment of risk to identify mothers for retention at health facilities until delivery. For mothers in their fourth trimester with transport challenges were retained until delivery, based on health worker’s assessment of timely arrival at the facility to avoid delays in reaching the facility.

*like today we have a woman from the islands whom we are accommodating in the labour ward waiting to deliver.* ***PF_HCIV_003_MW***

Delivery by caesarian section depended on the ability to pay for services by the mothers and whether the particular cases required blood transfusion. It enabled frontline health workers determine which cases to attend. Whereas some facilities insisted on payment beforehand or referred out in case of inability to pay, in others, assessment helped determine cases to attend to especially ones that did not require a blood transfusion.

*because we have skilled staff who can easily detect something and you know in this hospital if everything fails we would rather do a c-section and then have a live baby and a normal mother than keeping you there.* ***PNFP_Hosp_002_IC_Mat***

#### Aligning available resources to assessed risk

The face to face interactions during assessments was characterized by the desire to secure the mother’s cooperation throughout care provision. Interactive group ANC according to age group where mothers engaged actively in health education and monitoring their pregnancies was one of the strategies. It particularly characterized service access for younger mothers while older ones continued receiving individualized antenatal care services.

*yes, its peer where mothers of the same age group are arranged into the same group and when they come at the facility they are involved in doing certain simple things like taking BP, discussing issues that affect them in the presence of a health worker in the presence of a health worker that can help them realize the interventions that are necessary in keeping their pregnancies health and their lives.* ***PF_HCIV_004_MO***

Information on the availability of complementary services crucial for prevention of stillbirth was in some cases offered in a differentiated approach where perceived risk informed which services a mother would be referred to. Mothers with perceived elevated risk such as history of stillbirth were occasionally directed to such services. Specifically, delivery of Family Planning services was common as well as emotional support whose quality was shaped by the limited skills of the health workers as acknowledged in the quote below;

*Sometimes when it’s hard for them [midwives] we involve the counsellors because for us the counselling we do is the midwifery part but [if] there are some social issues we have a social worker in the hospital whom we sometimes involve when issues are involving social issues after getting the medical counselling.* ***PNFP_Hosp_002_MW***

Not all mothers received the same quality of counseling when presented for care. The level of cooperation with the frontline health workers influenced the quality of counselling received by the mothers. Instances of withholding vital information or referral to other sources for management were cited in the case of un-cooperating mothers. Nonetheless no standard counselling package was reported whereby some health workers preferred to focus on the cause only when it fell more on the mother’s behaviours.

*at times these mothers feel it was the negligence of the health worker who handled her. At times they may not want to see that midwife again so at this time we engage the in-charge or someone senior or if it’s beyond we consult and engage the doctor to help us.* ***PF_HCIV_004_IC_Mat***

*When it comes to counseling after baby loss, I counsel her about the cause and I also emphasize that next time don’t allow to use the herbs just come to the hospital and be handled by qualified health workers.* ***PNFP_Hosp_002_IC_Mat***

### Rationing of services to fit existing resources

Some health addressed stillbirth by rationing information and services to fit existing resources. This was observed to be true in the context of inadequate resources to support policy requirements. Limiting choices of family planning methods offered was observed where conflicting goals between organizational values and policy objectives. Some religious-affiliated health facilities were restrained from offering modern Family Planning methods. In such instances the package delivered was reduced to counseling without provision of commodities or administering methods as exemplified in the following quote;

*for the case of family planning we have nothing to do as Catholics we are supposed to use natural family planning … … we just counsel on natural methods which people don’t believe they are effective [and] it becomes a bit of a challenge. Because we tell her to go home and use moon beads, she may not even know [how to use it] and you have to confirm [the effectiveness of] those family planning after 6 months so before she confirms she has already conceived* ***PNFP_Hosp_002_MW***

### Limited choice of family planning methods

Where supplies appeared inadequate, it prompted health workers to use their discretion to fulfill policy objectives by improvising to ensure that mothers received family planning services even when it was not their preferred method.

*she insists on that particular method which is not available at the moment we can suggest to her that since it’s the same type lets give you another injectable and when you come back you will get the FP method of your choice. So it may confuse them somehow because it is us who would have diverted them from what they came for* ***PF_HCIII_006_IC_Mat***

During the patient-health worker interface, an assessment of risk-informed the packaging of information and mode of delivery. Subsequent prioritization during service delivery witnessed women perceived at high risk of stillbirth being referred from lower to tertiary level health facilities. Even within facilities, information was packaged depending on perceived risk with high-risk mothers receiving more information from specialists while the rest continued receiving routine services.

*What we do here we have categories of ANC care. We have a nurse-led ANC, we have a Doctor-led ANC, and we have a specialist ANC. For the midwife-led ANC when [they] identify a mother that has risk factors that need to be seen by a doctor, they will have to refer them to a doctor. And for the Doctor-led ANC when they have a risk factor [that] can be referred to a specialist and in all those levels, when the mother is not able to pay for the specialist direct is referred from the midwife to the doctor to the specialist and they can have optimum care [without paying]* ***PNFP_Hosp_001_MO***

While receiving services, referred mothers would be attended to first before the ones who started antenatal care services from tertiary facilities. Besides, referred mothers perceived to be high risk would be closely monitored through shorter return intervals and availed with the specialist’s telephone contacts for quick consultations.

*some of the very high-risk mothers we usually give them shorter return dates and we work in such a way that we give these mothers sometimes personal contacts or we give them the toll-free line to call. We teach them about any danger signs that if you feel like this it’s better for you to call and find out or you come to the facility* ***PNFP_Hosp_002_MO***

### Demand for services

When facilities were overwhelmed by demand, frontline health workers aligned available human resources to manage high volumes especially during ANC clinics. Related challenges in such contexts include the inability by the health workers to dedicate ample time to manage each of the cases and hence improvise by resorting to selective implementation of policy aspects.

*like if you go to an HCIV there are like 200 mothers and there are four midwives and if one of them worked at night probably another one is going to work during the day so that means that we have two midwives so how are you going to evaluate these mothers comprehensively?* ***PF_HCIV_003_MO***

### Limited resources and skills

Delivery of services in the context of limited resources led health workers to expend varying policy aspects to the mothers. Emotional support after experiencing a stillbirth which ordinarily extend beyond counseling to include addressing other social issues such as gender-based violence.

*there is a gap when it comes to counselling of these mothers because these days the counseling which is common is HIV counselling. So when it comes to the counselling of these mothers it is difficult [both to] the health workers and even the mother.* ***DHMT_007_Nur_02***

Other factors related to limited resources context include lack of ample workspace. For mothers that experienced a stillbirth, the facilities lacked spaces for mothers to rest while permitting them to grieve and resorted to isolation rather than separation.

*ofcause if it was a c-section they will keep around for the healing to take place then we usually try to put them in an isolation side which doesn’t have mothers with babies because sometimes they go into a bit of depression here and there when they hear the babies cry.* ***PNFP_Hosp_002_MO***

### Conflicting organizational expectations

Whereas some programs were implementing Maternal and Child Health interventions, focus was more towards prioritization of maternal mortality reduction. Stillbirth only came in because of the interconnectedness of interventions to address risk factors for both.

*you see when you look at the indicators our main emphasis is addressing the maternal mortality rates but the issue or stillbirth has not been well advocated for because when you look at how our health facilities are assessed mainly they are focusing on reducing the maternal mortality rate … .. But even when we were being taught obstetrics and gynaecology the principle was that if you are caught in between losing the mother and the baby, you should struggle to save the mother.* ***PF_HCIV_003_MO***

### Adapted routine to fit implementation context

In light of the limited resources, efforts to maintain quality standards were evident. Strict use of partograph for active labour monitoring, conducting perinatal death reviews to identify gaps, dissemination of performance data internally for shared understanding, proper documentation and submission of data to the district and MoH were some of the examples. Follow up to ensure compliance was particularly observed as seen from the quotation below;

*So we have recommendations and make sure we implement the things we have agreed upon. Like if the issue is using the partograph we will look at how you are using the partograph.* ***PNFP_Hosp_002_IC_Mat***

Streamlining and improvement of referral saw health workers from tertiary facilities focusing on a particular geographical area or specific lower-level facilities known to more often withhold mothers for long leading to late referrals.

*when we look at where these mothers are referred from commonly, there was a time when most of the referrals were coming from a certain health facility, so we had to go to that health facility and we had to talk to those people.* ***PNFP_Hosp_002_MO***

Counselling mothers after a stillbirth was reported to be a daunting task by the midwives. In circumstances where failure in convincing the mother about the possible causes was anticipated, such cases would be referred to the medical officers. It was reportedly conducted in a phased manner where midwives would start off with the emotional support and then refer to the medical officer to clarify circumstances leading to a stillbirth.

*What we do sometimes we fail but we try to explain to them some will understand but others will not. So for us midwives we don’t go there we send doctors who are firm because she will start asking now what happened? So if you are a midwife who is a bit timid you will start saying things which are not true,* ***PNFP_Hosp_002_IC_Mat***

## Discussion

### Main findings

This study aimed to describe how frontline health workers implemented strategies to address stillbirth risks at sub-national level final point of service delivery. Our results found that frontline health workers prioritised policy aspects in alignment with available resources to suit service delivery through offering differentiated care to mothers and by packaging of appropriate information and services based on context [14]. Through these strategies, it emerged that frontline health workers played an important role in determining which policy aspects got implemented. A key lesson observed across the health facilities was that translation of policy objectives to address stillbirth risk did not come as a single standalone package but rather overall pursuit of policy goals expressed by Ministry of Health. Strategies were rather integrated into ongoing policy alternatives currently running in Reproductive Maternal, Adolescent, Newborn and Child Health services. Where cases were fitting into the available resources, health workers proceeded to manage them as part of the routine services they offered. As seen from the results, although it was not an “either-or” scenario while translating policy recommendations, some aspects were prioritized more than others. Specifically, interventions that were already grounded in ongoing activities were easily adopted compared to those that were being introduced in the system specifically to respond to stillbirth.

### Prioritization of health service delivery to address gaps

From this study, the ability to identify gaps in the system that may contribute to inadequate care to avert stillbirth led health workers to devise strategies that saw the prioritization of service delivery in response to identified gaps. This is in pursuit of MoH policy priorities that are in line with the recommendations [5] for aligning health systems interventions to available resources in an integrated approach along the continuum of care from pre-pregnancy to postnatal health. A systematic review on the subject has documented evidence in support of appropriate spacing between pregnancies which is associated with better maternal and fetal outcomes [15]. Specific to stillbirth, literature has documented the causal relationship between short and long birth intervals on the risk of stillbirth [16]. Interventions to address birth spacing in the country are delivered through family planning programs. Besides, it was observed from this study that health workers in some facilities opted to introduce family planning at all entry points into care. This was in line with the MoH strategy of the provision of Reproductive Maternal Adolescent and Child Health services along the continuum of care [10]. The same has been emphasized elsewhere as being crucial in linking service delivery since stillbirth causes overlap. By health workers adapting strategies to health system context of limited resources, it can help maximize available human resource and improve cost-effectiveness [5].

One interesting finding from this study was that prioritization of service delivery aspects would sometimes extend beyond the formal policy provisions.. This was particularly the case when frontline health workers thought it was in their best interest to deliver services while protecting their reputation. Exemples included scenarios that reflected health workers conducting “mini-perinatal death audit meetings” ahead of the official one conducted by the facility MPDSR committee. As a quality improvement activity aimed at ensuring identification of health systems gaps that led to a stillbirth and make adjustments for implementation to avoid similar instances in future. Perinatal death audits are policy objectives that have been reflected in National guidelines with procedures for conducting one stipulated [17]. Cases were reported from this study where it was highly prioritized by the frontline health workers who held brainstorming session amongst those that handled a particular mother to establish the cause. Discussion of a high profile cases among staff outside formal proceedings is a universal practice around the world. Sometimes it occurs as a contribution to understanding the problem and contributing to solutions. This informal process is a key ingredient for quality and process improvement. It may also have occured due to fear of repercussions in case the gap fell on the health worker during the audit processes. This is understandable as a previous scoping review on the same attributed the low uptake of MPDSR to fear of the potential for litigation from families [18]. In the context of the study setting, not all health workers that handled the mother get invited during the audit committee meeting but rather representatives from the maternity unit usually the in-charge and the health worker that conducted the final delivery. The findings compare well with results from a study conducted in Jordan [19] that showed that health workers expressed a need to be part of the perinatal audits with reservations about the whole idea due to fear of having a conflict with the bereaved family.

Improvements in data capture, a key element highlighted from the global campaigns was also prioritized at the national level [20]. Several strategies are identified in national guidelines such as the spatial distribution of stillbirth burden [21], reflecting stillbirth as a district level and hospital performance indicator [22], perinatal mortality notification and review as a quality improvement indicator [17] among others. Another important finding from this study was that even at the facility level, health workers made efforts to ensure that stillbirths were among the indicators reported during daily handover reports from the maternity unit. Efforts were in addition to notifications, review and District health Information Systems (DHIS2) reporting procedures recommended from policy. In turn capacity gaps contributing to this trend would be identified and targeted skills building initiated. The results re-echo similar sentiments from elsewhere [23] calling on actors to count stillbirth to improve care for women which corroborate with World health organization (WHO’s) call for the same [24].

### Differentiated care to align with existing resources

Another important finding from this study was that depending on the perceived risk of stillbirth a mother possessed, differentiated care was offered by frontline health workers during antenatal care. It is a universal practice to This is understandable given that care during pregnancy is essential for identifying and modifying risk factors related to stillbirths like risky behaviours such as alcohol consumption and cigarette smoking [25]. Besides, it is during pregnancy care that micro-nutrient deficiencies can get addressed in addition to the identification and prevention of infections [26]. In some facilities, mothers with elevated risk such as hypertensive and diabetic disorders were referred to continue services from Medical officers while those with minimal risks received services from midwives during their routine visits. Lower level facilities without such cadreship were referred to tertiary facilities to be seen by a Doctor. Dialogue on birth preparedness included options for shorter return intervals, retention at a facility closer to due date or even delivered by caesarian section. These results are in agreement with findings from a modelling study that established the effect of managerial interventions in the reduction of stillbirth mortality [27, 28] reflecting high returns for delivery by caesarian section for mothers exceeding 42 weeks.

From the results, it emerged that emotional support through counselling mothers that had experienced a stillbirth was particularly challenging [29]. More than the additional resources, health workers informally recognize time as an essential element of resolution that no additional immediate resources such as staffing could probably substitute. This made health workers offer a differential package among mothers with a stillbirth and ones with a live baby. The former were encouraged to initiate family planning before discharge and the letter advised on appropriate spacing of approximately 2 years. Even among those that experienced a stillbirth, the packages depended on their levels of cooperation throughout the process with the uncooperative ones receiving minimal emotional support. In addition to family planning counselling, they had to explain the circumstances surrounding the loss [30]. The lack of trust and unfamiliarity between mothers and provider are certainly a handicap in determining grief comfort. of the other reason given was the inadequate counselling skills which made it impossible to comfortably manage the grieving mothers who oftentimes blamed health workers for the loss [31]. Similar results have been reported elsewhere [32] where difficulties were attributed to inadequate training. Health workers again noted that counseling was complicated by lack of space to conduct the sessions thereby ending up counseling the grieving mother from maternity wing among ones with live babies. Our findings corroborate with results from a systematic review [12] where midwives experienced stressful, emotionally challenging, uncomfortable and unprepared to care for bereaved families [33]..

### Rationing of services in the context of resources

Our findings show that in the absence of adequate resources to support the implementation of policy provisions to address stillbirth risks, health workers rationed services to suit resource availability. This was subject to their understanding and interpretation of policy requirements given the resources at their disposal. The availability of specific Family Planning methods at particular points in time affected access and utilization of family planning services and yet it has the potential to reduce stillbirth [34]. This was sometimes due to general stock-outs, partial stock out of particular commodities on specific days, or absence of health workers to administer certain methods. Prospects of a reduction in stillbirth rates, through fertility reduction leading to mortality reduction by scaling up family planning, have been documented [16]. However, the observation from this study that commodities may not be readily available implies that such benefits cannot be realized under such conditions.

The level of demand for available services made it challenging for health workers to accord each of the mother’s adequate time to manage. Overwhelming demand which created high patient loads has been associated with compromised quality in services delivered. To address this, instances were noted where mothers received services through group antenatal care where peer learning and counselling were encouraged [21]. The same extended to implementation of prioritized strategies to address stillbirths. Discretion, therefore, became one of the tools at the frontline health worker’s disposal to get around high volume especially during the ANC clinics which made them ration services. Rationing of information was common during these interactions where the assessment of risk informed the amount of information and mode of delivery. Limited skills also influenced what aspects of the policy to prioritize such as counselling skills not optimal.

### Limitation

The main limitation of this study was that it was based on a single district case. We acknowledge that implementation contexts vary significantly and may lead to different outcomes when it comes to the influence of frontline health workers on policy implementation processes. Therefore, the results and conclusions from this study need to be interpreted with caution in other settings. We aimed at an in-depth understanding of how the frontline health workers translated policies at the frontline to respond to stillbirth mitigation measures. Maternal and child health policy implementation in Uganda was at varying degree depending on service provision level and the capacities to offer the same. Although our sample represented all these service delivery levels including a mix of Public and Private-for-profit entities save for the Regional and National referral hospitals, we are unable to account for the contextual factors that may influence the health worker’s exercise of discretion in translating these policy intentions. Also, this study was unable to interview service users to get their perspectives with regards to the way frontline health workers exercised discretion while delivering services to them which would have helped in the triangulation of views expressed by the later. Besides, the adoption processes of the policy priorities to respond to stillbirth were still evolving and likely to play out differently in different contexts by the time field data collection was conducted. None the less this study had some strengths. Specifically, it contributes to filling of the knowledge gap with regards to implementation experiences of frontline health workers following global campaigns which called upon governments to prioritize interventions aimed at addressing stillbirth by 2035. The provision of context and the contributing factors likely to have led frontline health workers to adjust aspects of policies at the final point of implementation will likely provide lessons on how best to inform policy implementation. It will provide policymakers and implementers with insights of what needs to be in place if policy intentions are to be realized. The results presented in this study are drawn from service provision following the routine standard of care and not pilot projects. Such a setting is not contaminated with external factors likely to sway outcomes and therefore good to provide key implementation lessons in a real-life setting. Whereas some of the stillbirth global campaign priority areas had been implemented in a project mode with uncertain outcomes, by the time of the study were rolled out into routine standard of care. Our results are from the real world implementation context and therefore likely to have practical implications since the context may not differ much.

## Conclusion

In light of the study findings and ensuing discussion, frontline health workers were seen to align resources to tailor implementation of aspects from the policy provisions. It was influenced by their understanding of risk, feasibility of implementation and the desire for optimal health systems performance. Our analysis indicated that the interplay of contextual factors at the final point of policy translation into service delivery such as the health system capacities, the commitment to achieve policy objectives, alignment of available resources to policy targets in an integrated manner ought to be considered while setting policy objectives.

## Supplementary Information

**Additional file 1.**

## Data Availability

The datasets used and/or analysed during the current study are available from the corresponding author on reasonable request.

## Abbreviations

ANC: Antenatal care
AHSPR: Annual Health Sector Performance Report
ART: Antiretroviral Therapy
CME: Continuous Medical Education
CSO: Civil Society Organizations
DOT: Directly Observed Therapy
DHT: District Health Team
DHMT: District Health Management Team
DHIS: District health Information Systems
HBB: Helping Babies Breath
ICCM: Integrated Community Case Management
LMIC: Low and Middle-Income Countries
MPDSR: Maternal and perinatal Death Surveillance and Review
MoH: Ministry of Health
VHT: Village Health Team
