# Supplementary material for: Prioritization of interventions in pursuit of maternal health policy objectives to mitigate stillbirth risks. An exploratory qualitative study at subnational level in Uganda
Source: BMC Health Serv Res. 2021 Jan 11;21:53. doi: 10.1186/s12913-020-06046-z (PMC7802206; doi:10.1186/s12913-020-06046-z)
Supplement: Supplementary file 1 — Additional file 1. [file 12913_2020_6046_MOESM1_ESM.docx]

## Additional file 1: KII guide: Subnational level implementation of Maternal and Child Health policies to address stillbirth.

1. Would you kindly discuss with us your experience with implementing maternal health policies to address stillbirth at this health facility
   1. Describe your experience following rules/guidelines or using discretion in implementing maternal health policies at this health facility
   2. Tell me more about the aspects that make you describe yourself as such, more so when you are providing ANC and delivery services.
2. What has been the role of health facility management in influencing health workers while implementing maternal health policies to address stillbirth
   1. Can you give me an example of what aspects the management has an influence over health workers
   2. Tell me more about the extent to which they exert their influence
   3. Comment on the aspects where management leave health workers to be in charge while implementing maternal health policies
   4. Tell me more about the extent to which they exert their influence
   5. Can you tell me more about the resources availability and in what ways they limit or favour health workers use of discretion?
   6. Tell me more about some of the health facility management practices that may influence the frontline health workers’ practices in translating policies
3. Would you elaborate on the role of the health workers’ social demographic characteristics and how it influence the way they implement maternal health policies to address stillbirth risks at lower level health facilities
   1. Tell me more about the gender of the health workers
   2. How about the age of the health worker
   3. How about the education level
4. In what ways does the workplace related characteristics influence the health workers on how they implement maternal health policies to address stillbirth at lower level health facilities
   1. Tell me more about the time spent in that particular health facility
   2. How about the overall work experience (seniority)
   3. Tell me more about the cadre ship of the health worker (nurse, midwife, clinical officer, doctor)
   4. How about the health worker’s welfare (stay at facility or away in community)
   5. Tell me more about the familiarity with policy objectives/goals
5. Does the mother’s characteristics have any influence on how health workers implement maternal health policy to avert stillbirth?(how each of these affects health workers exercise of discretion)
   1. Tell me more about the social status
   2. How about the economic status (ability to effect referral or let them stay)
   3. What about the maternal age
   4. Tell me more about the total pregnancies
   5. How about the birth interval
   6. What about the ANC attendance
   7. Tell me more about the social support from spouse and family
6. In your opinion what is the influence of the health workers’ perception of still birth risk on their implementation of maternal health policies to mitigate the same at lower level health facilities
   1. Tell me more about the mothers perceived to be at high risk of stillbirth
   2. What about those mothers perceived to be at low risk of stillbirth
7. What has been the influence of policy enforcement strategies on the frontline health workers’ implementation of maternal health policies to mitigate stillbirth risks?
8. How about the influence of contextual factors ie availability and unavailability of resources (human resource, supplies and commodities, health facility infrastructure)
